# Antinociceptive and Anti-Inflammatory Activities of *Bridelia retusa* Methanolic Fruit Extract in Experimental Animals

**DOI:** 10.1155/2014/890151

**Published:** 2014-11-17

**Authors:** Tekeshwar Kumar, Vishal Jain

**Affiliations:** University Institute of Pharmacy, Pt. Ravishankar Shukla University, Raipur, Chhattisgarh 492 010, India

## Abstract

Antinociceptive and anti-inflammatory potentials of methanolic extract of *Bridelia retusa* fruit (BRME) were evaluated against different animal models in rodents. Antinociceptive effects of BRME were assessed in mice using the acetic acid-induced writhing and formalin test. Anti-inflammatory effects of BRME in three different doses, namely, 100, 200, and 400 mg/kg, were evaluated by utilizing different animal models representing various changes associated with inflammation, namely, carrageenan-induced paw oedema, histamine and serotonin-induced paw oedema, arachidonic acid-induced paw oedema, formalin-induced paw oedema, TPA-induced ear oedema, acetic acid-induced vascular permeability, total WBC count in paw fluid, and myeloperoxidase assay. Also BRME was phytochemically evaluated using chromatographic method. The BRME did not exhibit any signs of toxicity up to a dose of 2000 mg/kg. The extract showed statistical significant inhibition of induced nociception and inflammation in dose dependent manner. The higher dose of extract significantly inhibited pain and inflammation against control (*P* < 0.001). HPLC results revealed the presence of gallic acid and ellagic acid as phytoconstituents in BRME and it was proven as anti-inflammatory agents. The present study scientifically demonstrated the antinociceptive and anti-inflammatory potential of fruit of *B. retusa* methanolic extract. These effects may be attributed to the presence of polyphenolic phytoconstituents in the extract.

## 1. Introduction

In recent past a tremendous aggressive trust has been observed in natural origin as a prime source of healthcare. This fact significantly contributed in the development of modern medicine of natural origin. The uses of natural medicines are widespread and plants still present a large source of structurally novel phytocompounds that might serve as leads for the development of novel pharmaceutical agents for the prevention and treatment of diseases and ailments [1].

The biodiversity culture of India is very wide and several ethnic plants have a prolific traditional usage with great phytotherapeutic prospective. About 1500 species of medicinal plants are available in India, even though many of the ethnically used plants lack scientific validation so far. Ethnic medicine has come to be an inimitable source of knowledge of medicinal plants and their therapeutic uses with clues for future scientific investigations, which usually confirm the legitimacy of their usage [2].

Recently medicines of plant origin have received great deal of attention compared to synthetic ones because of their affordability and accessibility due to their potent antioxidant activities, lesser side effects, and pecuniary feasibility. Today, researcher have been paying attention on consumption of medicinal plants derived natural products such as polyphenols, flavonoids, coumarins, steroids, terpenes, alkaloids, and many more phytogroups as food and nutraceutical stuff to protect the body as well as foods against various oxidative damages. Several anti-inflammatory, hepatoprotective, antidiabetic, antinecrotic, neuroprotective, and anticancerous drugs have recently been shown to have an antioxidant mechanism as part of their activity. Hence, the modern drift is to conduct investigations on ethnopharmacological remedies [3]. Inflammation is a nonspecific immunological defense mechanism and is triggered by mechanical injuries, microbial infections, burns, allergens, and other noxious stimuli. Harmful stimuli lead to activation of nociceptors through the release of variety of chemical mediators, such as excitatory amino acids, vasoactive amines (histamine, serotonin), proteins, peptides, nitric oxide (NO), arachidonic acids (prostaglandins E2, leukotrienes), and cytokines [TNF-*α* and interleukin-1], which act on specific receptors and ion channels, contributing to the induction of pain and inflammation [4].

These mediators initiate many complex arrays of processes such as activation of many enzymes, release of several mediators, cell migration, extravasations of fluid, increase of protein denaturation, and membrane alterations. Increase in vascular permeability is implicated in the pathogenesis of many diseases including excessive inflammation diseases like rheumatoid arthritis, asthma, periodontitis, inflammatory bowel disease, atherosclerosis, Alzheimer's disease, cancer, diabetes, neurodegenerative, cardiovascular, and other life-threatening and debilitating diseases [5]. Therefore, these proinflammatory mediators are important targets in inflammation.

Many nonsteroidal anti-inflammatory drugs (NSAIDs) are the most widely prescribed therapeutics, mainly for the treatment of pain and inflammation. However, long-term clinical usage of NSAIDs is associated with significant side effects, such as gastrointestinal lesion, bleeding, and peptic ulcer [6]. Hence, as an alternative, ethnopharmacological remedies are getting an increased therapeutics market share due to their better action and fewer adverse effects.

Natural polyphenols (gallic acid and ellagic acid) have showed antimutagenic, anticancer, antioxidant, and anti-inflammatory activities [7, 8] and flavonoid inhibited nociceptive behavior and inflammatory response in animals [9].

*Bridelia retusa* Spreng. Syn:* Bridelia airyshawii* (Family-Euphorbiaceae) is found throughout hotter parts of India [10]. Chemical and pharmacological studies of different parts of* Bridelia retusa* have revealed the presence of sesquiterpenes, triterpenoids, flavonoids, and phenolic compounds [11] as well as wide variety of pharmacological activities including antibacterial, antifungal, antimalarial, antiviral, antidiarrhoeal, anticonvulsant, antianemic, antidiabetic, antinociceptive, and anti-inflammatory activities [12–14]. The fruit pulp and seeds contain gallic acid, ellagic acid, and *β*-sitosterol [15].

Our literature survey revealed that there is no previous work approach that has been made for fruits of* B. retusa*. Therefore, the present investigation was undertaken to evaluate the antinociceptive and anti-inflammatory activity of fruit extract of* Bridelia retusa* using various in vivo models.

## 2. Materials and Methods

### 2.1. Collection and Identification of Plant Material

The plant fruits of* B. retusa* were collected from forest, managed by Government of Chhattisgarh State Forest Division in January, 2014. The collected plant was botanically recognized by Dr. Shyam Biswa. A voucher specimen (CNH-2014/RSU/TV-02) was submitted to the Central National Herbarium, Hawrah, India.

### 2.2. Preparation of Fruit Extracts of* B. retusa*

*B. retusa* fruit was dried at 40–45°C, ground to powder, and extracted (10 g) with methanol by buchi speed extractor (pressure 100 bar, temperature 100°C, heatup time 5 min, hold time 3 min, number of cycles 5, time to cycle end 5 min), and extraction was repeated three times. The extract was mixed and concentrated under reduced pressure below 40°C using rotary evaporator (Ika RV 10). The dried crude extract (yield ratio 58.3%) was maintained at 4°C for further use.

### 2.3. Chemicals

Acetonitrile, acetic acid and methanol (HPLC grade), arachidonic acid, 12-O-Tetradecanoylphorbol 13-acetate (TPA), N,N-dimethylformamide, and indomethacin were procured from Sigma-Aldrich, Pvt. Ltd. All the other chemicals and solvents of standards analytical grade were obtained commercially.

### 2.4. HPLC Analysis

#### 2.4.1. HPLC Conditions

The phytochemical characterization of BRME was conducted by high performance liquid chromatography (HPLC) analysis using autosampler Water 2459 series. Separation was achieved at 40°C on a 250 mm × 4.6 mm i.d. 5 *μ*m, C18 column (Water sun fire column). The mobile phase consisted of acetonitrile (Solvent A) and 0.2% acetic acid in water (solvent B) with the flow rate of 1 mL/min for 45 min. The elution was performed utilizing the following solvent gradient over a total run time of 45 min: 0 min, 95% B, 0–10 min, 90% B, 10–20 min, 60% B, 30–40 min, 50% B, and 40–45 min, 100% B. The solvent was pumped using 515 HPLC pump with a flow rate of 1 mL/min. Data analysis was performed using Water empower software.

The contents of gallic acid and ellagic acid of BRME were qualified and quantified in the HPLC assay. In qualitative analysis, comparisons were made with the retention time and maximum absorption of the standards whereas, in quantitative analyses, comparisons were made with peak areas under the standard curves.

#### 2.4.2. Preparation of Sample Solution

Fruits of* B. retusa* (10 g) were extracted by hydrolysis of sample with 30 mL of methanol under refluxed for 1 hour. Extract obtained was filtered using Whatman filter paper number 42. 10 mL of distilled water was added to the filtrate and evaporated to a volume of 10 mL. Samples were analyzed immediately after extraction in order to avoid possible chemical degradation [16].

### 2.5. Animals

Wistar rats (150–250 g) and Albino mice (25–35 g) of both sexes were used for studies. The animals were obtained from animal house of Rungta College of Pharmaceutical Sciences and Research, Bhilai, Chhattisgarh, India. The animals were acclimatized in the pharmacology research laboratory, for seven days before the start of experiments. The animals were kept in groups of eight per cage under standard environmental conditions (12 : 12 h light : dark cycle at 25 ± 2°C) and were provided with standard pellet diet and water given ad libitum. Food was withdrawn 12 hours before experiments. The studies were approved by the Institutional animal ethics committee (1189/PO/a/08/CPCSEA) and were performed as per the guidelines of Committee for the Purpose of Control and Supervision of Experiments on Animals (CPCSEA), Chennai, India.

### 2.6. Acute Toxicity Study

It was carried out as per the OECD guidelines 420. The animals were administered orally with the different doses of extract. Female Wistar rats weighing 150–180 g were used for the study. The animals were continuously observed for the autonomic and behavioral changes for 12 hrs and the mortality was observed for 24 hours. No mortality was found up to a dose of 2000 mg/Kg which was taken as the end point dose till the completion of 24 hours. The doses of 100 mg/kg, 200 mg/kg, and 400 mg/kg were selected for the further activity on the basis of pilot studies performed at our lab (data not shown).

### 2.7. Antinociceptive Studies

#### 2.7.1. Acetic Acid-Induced Writhing Test

The acetic acid-induced writhing test was performed as per the method described by Collier et al. [17] with slight modifications. Mice of either sex were randomly divided into five groups (*n* = 6). The drugs and solvent treatment was same as in above mentioned studies. 30 min after treatment, each mouse received acetic acid solution (1% acetic acid of 10 mL/kg body weight) intraperitoneally. The animals were then kept in transparent cages individually for observation of writhes. A single contraction of abdominal wall with stretching of hind limb was considered as one writhe. Each animal was observed separately for a period of 5–30 min immediately after the administration of acetic acid. % inhibition of writhing was calculated using the following formula:

(1) $$\begin{array}{c} \%\;\text{inhibition}\;\text{of}\;\text{writhing}=\left[1-\frac{W_{T}}{W_{C}}\right]\times 100, \end{array}$$

where *W*_*C*_ and *W*_*T*_ represent the mean number of writhing of control group and test group, respectively.

#### 2.7.2. Formalin Induced Nociception Test

The antinociceptive effect was also carried out using the formalin test [18]. Rats were injected with 20 *μ*L of 5% formalin (in 0.9% saline) solution into the dorsal surface of the right hind paw, 1 h after oral administration of BRME (100, 200, and 400 mg/kg) or indomethacin (10 mg/kg). Control group received 5% formalin. The time spent licking the injected paw was observed for 30 min soon after the injection of formalin was recorded and expressed as the total licking time in the early phase (0–5 min) and late phase (15–40 min) after the formalin injection. Antinociceptive effect was indicated by the reduction of the total mean time determined for the test groups compared to the control group.

### 2.8. Anti-Inflammatory Activity

#### 2.8.1. Carrageenan-Induced Rat Paw Oedema

This was determined as described by Winter et al., [19]. The Wistar rats (weighing 110–190 g) were used in this study divided into five equal groups (*n* = 6). Group-1 received distilled water (10 mL/kg, p.o.) (Control), group-2 received indomethacin (reference drug 10 mg/kg, p.o.), and groups 3–5 received extracts (100, 200, and 400 mg/kg in 1 mL distilled water, p.o.), respectively. After 30 minutes, acute inflammation was produced in the right hind paw of each rat by subplantar injection of 0.05 mL freshly prepared carrageenan suspension (1%) in normal saline. The volumes of the oedematous paws were measured using Plethysmometer (PLM 01 PLUS (Orchid scientific, India)), following oral administration of the plant extract/standard, 0 min (before carrageenan injection) and at every 1 h intervals for 5 h. Oedema was expressed as the increment in paw thickness due to carrageenan administration [19].

The percentage of anti-inflammatory activity was calculated using the formula given below:

(2) $$\begin{array}{c} 100\times \left(1-\frac{V_{t}}{V_{c}}\right), \end{array}$$

*V*_*c*_ is control group mean; *V*_*t*_ is test group mean.

#### 2.8.2. Histamine and Serotonin-Induced Rat Paw Oedema

The method of Amann et al., [20] was adopted for paw oedema measurement. Rats were divided into five groups (*n* = 6) treated with distilled water (10 mL/kg, p.o.), indomethacin (10 mg/kg, p.o.), and* Bridelia retusa* (100, 200, and 400 mg/kg, p.o.). After 1 h of oral administration, paw oedema was induced in rats by subplantar injection of 0.1 mL of freshly prepared histamine (0.001 mg/mL) or serotonin (0.001 mg/mL) solutions, respectively, in the right hind paw of the rats and paw oedema was determined. The thickness of the paw was measured at 60 min interval for 5 h after the injection of histamine or serotonin:

(3) $$\begin{array}{c} \%\;\text{inhibition}=100\times \left(1-\frac{V_{t}}{V_{c}}\right), \end{array}$$

where *V*_*c*_ represents mean oedema in control and *V*_*t*_ means oedema in group treated with standard/extract.

#### 2.8.3. Arachidonic Acid-Induced Paw Oedema in Rats

The assay was performed as described by Di Martino et al. [21] with slight modification. Rats of either sex were divided into 5 different groups (*n* = 6 each). Rats were treated intraperitoneally with saline (control) or indomethacin (10 mg/kg) or BRME (100, 200, and 400 mg/kg). After 30 min of treatment, 100 *μ*L of arachidonic acid (0.5%) was administered into the subplantar side of the right hind paw of each rat and the oedema volume was determined at 1 h interval for 5 h.

#### 2.8.4. Formalin-Induced Mice Paw Oedema

The formalin-induced inflammation test was conducted based on the method of Turner [22]. Animals were randomly divided into five groups (*n* = 6). The inflammation was produced by subplantar injection of 20 *μ*L of freshly prepared 2% formalin in the right hind paw. The extracts at doses of 100, 200, and 400 mg/kg or indomethacin 10 mg/kg and given distilled water (10 mL/kg, p.o.) were given 1 h prior to formalin injection. The daily changes in paw oedema were measured 1 h prior to and also after drug treatment using vernier caliper each day. The drug treatments were continued for 6 repeated days:

(4) $$\begin{array}{c} \%\;\text{inhibition}=100\times \left(1-\frac{V_{t}}{V_{c}}\right), \end{array}$$

where *V*_*c*_ represents mean oedema in control and *V*_*t*_ means oedema in group treated with standard/extract.

#### 2.8.5. TPA-Induced Mouse Ear Oedema/12-O-Tetradecanoylphorbol-13-acetate- (TPA-) Induced Mouse Ear Oedema

The animal inflammation model for ears oedema using TPA in mouse has been previously described by de Young et al., [23]. Each experimental group orally received the vehicle, extract (100, 200, or 400 mg/kg), or indomethacin (1 mg/kg). All treatments were dissolved in acetone and applied topically on both ears after the administration of TPA. The left ear (blank) received the same volume of solvent acetone. After 1 h, TPA (2.5 *μ*g) as phlogistic agent dissolved in acetone (25 *μ*L) was topically applied on both anterior and posterior surface of the right ear and 4 h later the animals were killed by cervical dislocation, and a section (6 mm diameter) of the central portion of both ears was obtained and weighed. The thickness of the ears was measured before and at 4 hours after induction of inflammation and the degree of ear swelling was calculated using expressed as the increase in ear thickness (mm). Inhibition percentages were calculated by comparison with the control group:

(5) $$\begin{array}{c} \%\;\text{Inhibition}=\left(\Delta T_{\text{control}}-\Delta T_{\text{treatment}}\right)\times 100, \end{array}$$

where Δ*T* = *T*_*t*_ − *T*_*nt*_; *T*_*t*_ is the thickness of the section of the treated ear; *T*_*nt*_ is the thickness of the section of the nontreated ear.

#### 2.8.6. Acetic Acid-Induced Vascular Permeability

This test was carried out by the method described by Whittle [24] with a slight modification. 5 groups of mice (*n* = 6 each) were used for the study. Group I served as vehicle control, groups II, III, and IV were treated with extracts 100, 200, and 400 mg/kg, respectively, whereas group V received indomethacin 10 mg/kg orally. One hour after the treatment, mice were injected with 1% acetic acid solution (10 mL/kg) in saline intraperitoneally. Just after 2% Evans blue solution (10 mL/kg) in normal saline was injected intravenously through tail vein. 30 min later, mice were sacrificed by cervical dislocation and the peritoneal cavity was washed with normal saline (5 mL). The washings were collected and made volume up to 10 mL into heparinized test tubes and subsequently centrifuged at 2,000 rpm for 10 min. The dye content in the supernatant was measured spectrophotometrically (Shimadzu 1800, Japan) at a wavelength of 610 nm. The vascular permeability effects were expressed as the total amount of dye leaked into the intraperitoneal cavity based on their absorbance.

#### 2.8.7. Total WBC Count in Paw Fluid after Acute Inflammation

Total WBC count in paw fluid was executed as explained by Prempeh and Mensah-Attipoe [25] with slight modification. 30 rats were divided into 5 groups (*n* = 6). Group I served as vehicle control, groups II, III, and IV were treated with extracts 100, 200, and 400 mg/kg, respectively, whereas group V received indomethacin 10 mg/kg orally. 1 h later, inflammation was induced by injecting 0.1 mL carrageenan (1% w/v in normal saline) into the subplantar surface of the hind paw of all rats. 3 h after the administration of the inflammatory agent, the plantar aponeurosis of the inflamed paw was injected with 2% xylocaine, incised and the paw fluids of each rat aspirated (using 26G hypodermic needle) and slowly spurted into a test tube. The residual fluid was gently squeezed out, ensuring that blood did not mix with the fluid.

Take 0.02 mL of paw fluid in a test tube and add 0.38 mL of WBC fluid (3% acetic acid with crystal violet dye) and mix well. The mixture was transferred into a counting chamber, and the total number of WBCs is counted under a microscope (×40).

Total number of WBCs = number of cells counted × depth factor (10) × dilution factor (20) × area factor (0.25).

The inhibition of migration of WBC was evaluated as percent reduction/inhibition in the treated animals relative to control animals using the relation:

(6) $$\begin{array}{c} \%\;\text{inhibition}\;\text{of}\;\text{WBC}\;\text{migration}=100\times \left(1-\frac{T}{C}\right), \end{array}$$

where *C* is the total WBC in control group and *T* is the total WBC in treated group.

#### 2.8.8. Myeloperoxidase Assay

Ear tissue for MPO assay was obtained by previously described method of de Young et al., [23] and assay was carried out with minor modification of the method described by Bradley et al., 1982 [26]. Each ear section was placed in an Eppendorf tube with 1 mL of 80 mM phosphate-buffered saline (PBS, pH 5.4), containing 0.5% hexa-decyl-trimethyl-ammonium bromide (HTAB), and then homogenized (45 s at 0°C) and decanted into a microfuge tube. Tube was washed with a second 0.75 mL aliquot of HTAB in buffer and wash was added to the tube and the 1.5 mL sample was centrifuged at 1250 rpm at 4°C for 15 min. The resulting supernatants (30 *μ*L in triplicate) were added to 96-well microtitre plates. For assay, 200 *μ*L of a mixture containing 100 *μ*L of 80 mM PBS (pH 5.4), 85 *μ*L of 0.22 M PBS (pH 5.4), and 15 *μ*L of 0.017% hydrogen peroxide were added to the wells. The reaction was triggered with the addition of 20 *μ*L of 18.4 mM 3,3,3-tetramethylbenzidine (dissolved in 50% aqueous N,N-dimethylformamide). Microliter plates were incubated at 37°C for 5 min after which the reaction was stopped by adding 30 *μ*L of 1.46 M sodium acetate (pH 3.0). The enzyme activity was determined by measuring the optical density using a plate reader (BIORAD 550.) at 630 nm and expressed as the inhibition percentage of MPO levels determined as the absorbance difference between the control group (vehicle) and the treated group compared to the absorbance observed in the control.

### 2.9. Statistical Analysis

The results were presented as a mean ± SEM of the indicated number of experiments (*n* ≥ 3). One-way analysis of variance (ANOVA) followed by Dennett's test or by two-way ANOVA by Bonferroni's test were used to find the statistical differences between control and tested groups. All statistical analyses were performed using GraphPad Prisms (Product version 5.03).

## 3. Results and Discussion

### 3.1. HPLC Analysis

**Figure 1:**
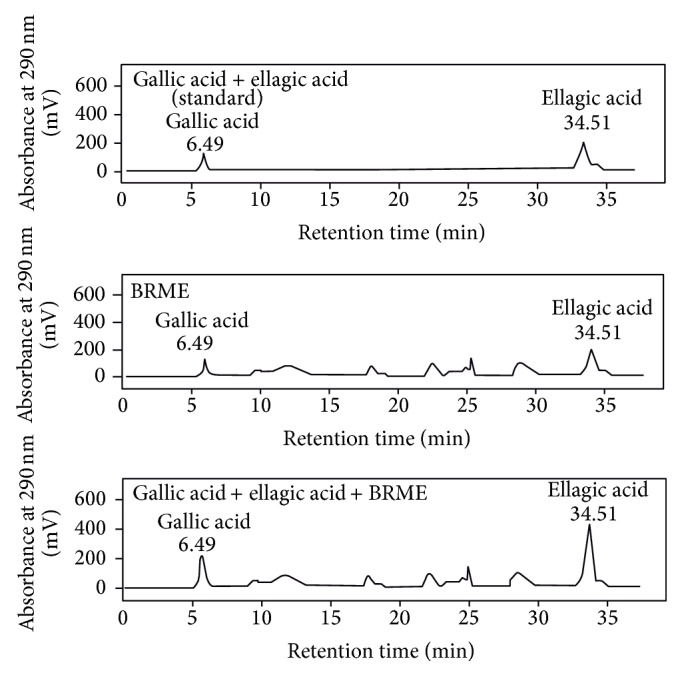
HPLC chromatogram of the standard gallic acid, ellagic acid, BRME, and mixture of standard + extract.

It has been anticipated that the compounds showing anti-inflammatory activity, present in* B. retusa* fruit extract, were phenolic compounds (gallic acid and ellagic acid). The phenolic compounds were detected using UV absorption spectra monitored at 290 nm showing good absorption. This method was employed to separate, identify, and quantify the phytoconstituents in the extract. The concentration was determined by calculating the HPLC peak areas which are proportional to the amount of analytes in peak and presented as the mean of the two determinations which was highly repeatable. The result indicated that a mixture of solvents was pumped in gradient mode that could be separate phenolic compounds in less than 45 min with agreeable peak resolution. There was a clear peak with the retention time of 6.49 and 34.51 min in the chromatographic analysis of the methanol extract, and a peak with a similar retention time was observed by HPLC analysis of standard gallic acid and ellagic acid. In a spiking experiment combination of standards with BRME (1 : 1) increased the peak area at the same retention time, suggesting that the peak similarly seen for standards is included in this extract (Figure 1). This HPLC method is simple, rapid, and accurate. In addition, gallic acid and ellagic acid should be considered as an indicative marker for standardization of* B. retusa.*

### 3.2. Analgesic Studies

#### 3.2.1. Acetic Acid-Induced Writhing Test

**Figure 2:**
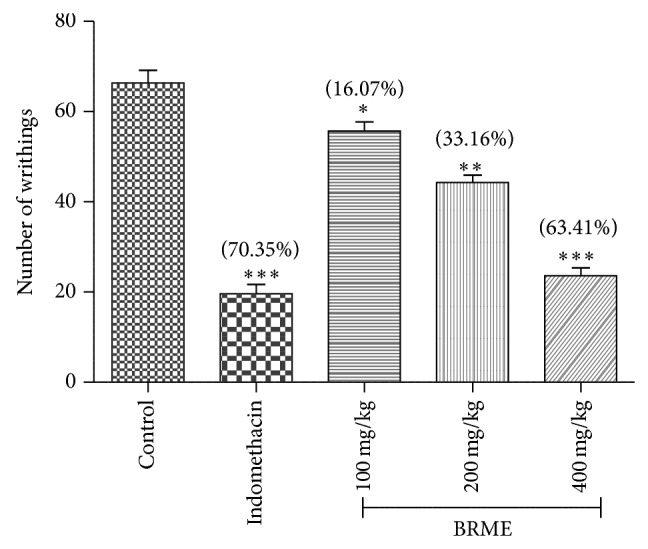
Effects of BRME and indomethacin in the acetic acid-induced abdominal writhing test in mice. Values are expressed as means ± SEM (*n* = 6). ^*^ *P* < 0.05, ^**^ *P* < 0.01, and ^***^ *P* < 0.001, compared to the control group.

The acetic acid-induced abdominal constriction test is widely used to study the peripheral antinociceptive activity of drugs in vivo [17]. This test is not suitable for selective pain test. Writhing test also bounces positive results with sedatives, muscle relaxants, and many other pharmacological activities which question its sensitivity and specificity. Intraperitoneal administration of acetic acid involved local peritoneal receptors and are mediated by indirect release endogenous nociceptive mediators, prostaglandins [27], and sympathomimetic system mediators like PGE2 and PGF2*α* that stimulate the nonsteroidal anti-inflammatory drugs (NSAIDs) and opioids-sensitive nociceptive neurons. Acetic acid also persuades sympathetic nervous system mediators that achieve peak level at first 30 min after acetic acid injection. In the same period of time, writhing is to be initiated due to excitation of pain in nerve endings and also activation the production of noxious substances within the peritoneum [28].* B. retusa* extract significantly decreases in the number of writhes at all the three doses in a dose-dependent manner as shown in Figure 2. The dose of 100 and 200 mg/kg showed reduction of 16.07% (*P* < 0.05) and 33.16% (*P* < 0.01), respectively, in the number of writhes. The dose of 400 mg/kg showed a reduction in number of writhes by 63.41% (*P* < 0.001) which was found to be comparable with the response of the standard drug, indomethacin (10 mg/kg) (% inhibition 70.35%, *P* < 0.001). The entire groups were compared to control group. The overall study suggests that the peripheral analgesic effect of the extract may be due to inhibition on synthesis or action on algogenic substances or the inhibition at the central level of the transmission of painful stimuli. Thus, peripheral analgesic effect is to be hindered by the anti-inflammatory compounds.

#### 3.2.2. Formalin-Induced Nociception

**Figure 3:**
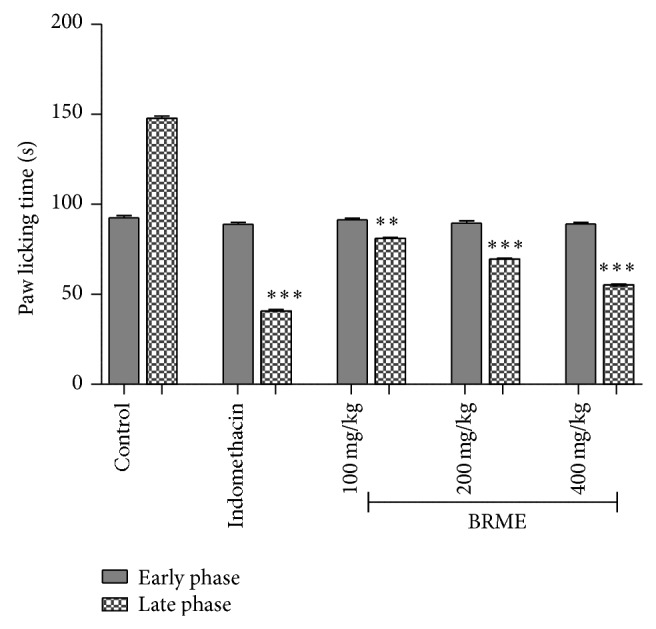
Effect of BRME and indomethacin on the licking induced by a formalin injection in rats. The total time spent licking the hind paw was measured in the early and late phases after intraplantar injection of formalin. Results are represented as mean ± SEM (*n* = 6). ^**^ *P* < 0.01, ^***^ *P* < 0.001, when compared to the control.

Formalin-induced nociceptive response gives a biphasic pain response and is very useful in assessment of pain relieving efficacy and mechanism of analgesic action of the test drug [29]. Two distinct phases constitute the formalin test. The first neurogenic pain normally peaked 5 min after formalin injection due to chemical stimulation of nociceptors of sensory afferent C-fibres and the second phase (15–30 min after formalin injection) representing the neurogenic and inflammatory pain responses, respectively, is mediated by a combination of NMDA and non-NMDA receptors in the peripheral input and spinal cord sensitization [30]. Centrally acting drugs such as narcotics inhibit both phases of formalin-induced pain while peripherally acting drugs inhibit only the second phase [4]. The formalin-induced pain test was used to find out the site of analgesic activity observed in the experimental extract. The results demonstrate that pretreated animal with BRME did not produce any significant antinociceptive effect in the first phase compared to control group. BRME at higher doses (200 and 400 mg/kg) and indomethacin (10 mg/kg) produce (Figure 3) marked (^**^ *P* < 0.01, ^***^ *P* < 0.001) reduction in the licking time in the second phase of the nociception. This suggests that extract against pain possesses analgesic effect possibly mediated through the inhibition of prostaglandin synthesis.

### 3.3. Anti-Inflammatory Activity

#### 3.3.1. Carrageenan-Induced Rat Paw Oedema

Carrageenan-induced oedema method was used for study of nonsteroidal anti-inflammatory drugs [31]. Oedema formation in the rat paw is a triphasic event with involvement of several inflammatory mediators. The initial phase (during the first 2 h after carrageenan injection) is attributed to the release of chemical mediators such as histamine and serotonin. The intermediate phase (2–2.5 h) of oedema is due to the release of kinin, protease, and lysosome. The last phase (2.5–6 h) just begins after the intermediate phase and is subsequent to the emancipation of bradykinin and prostaglandins such as PGE2 in tissue [32]. This phase is awfully sensitive to most clinically effective anti-inflammatory drugs.

Progression of oedema provoked by carrageenan is commonly correlated with the early exudative stage of inflammation and is useful phlogistic tool for investigating systemic anti-inflammatory pathology. Paw oedema volume has been increasingly used to test new anti-inflammatory drugs. The extract showed dose-dependent inhibitory activity in carrageenan-induced paw inflammation over a period of 5 h. This indicates inhibitory action for release of histamine and serotonin in early phase and arachidonic acid in later phases, producing an oedema dependent on mobilisation of neutrophils.

Cyclooxygenase plays an important role in conversion of arachidonic acid into prostaglandins in the later inflammation phase in the carrageenan-induced oedema model and this enzyme is considered to be a known target for a variety of NSAIDs such as aspirin [33].

The anti-inflammatory activity of BRME against carrageenan-induced paw oedema in rat revealed time and dose-dependent inhibition (Table 1). The results showed that the extract at 200 and 400 mg/kg exhibited statistical significant activity comparable to control. The maximum inhibition 42.5% was observed with 400 mg/kg of crude extract at 5 h, while the 200 mg/kg showed 32.8%. Standard indomethacin showed 63.98% inhibition at 5 h of drug treatment. Hence from our result, it is clear that BRME inhibits all the phases by inhibiting inflammatory mediators.

#### 3.3.2. Histamine and Serotonin-Induced Rat Paw Oedema

Histamine and serotonin are important inflammation mediators of acute phase of inflammation causing increase in vasodilatation and vascular permeability [34]. In this study all the doses of BRME effectively suppressed the oedema produced by histamine and serotonin, so it may be suggested that its anti-inflammatory activity is possibly backed by its antihistaminic activity. The BRME also effectively suppressed the inflammation produced by serotonin induced by hind paw oedema, which indicates that the BRME may exhibit its anti-inflammatory action by means of inhibiting either the synthesis, release, or action of inflammatory mediators, namely, histamine, serotonin, and prostaglandins that might be involved in inflammation. BRME produced significant (*P* < 0.05, 0.01, 0.001) dose-dependent inhibition of paw oedema development with the greatest effect produced at 5 h for 400 mg/kg 46.99% and 55.33% in histamine (Table 2) and serotonin (Table 3) treated group, respectively.

#### 3.3.3. Arachidonic Acid-Induced Paw Oedema in Rats

The anti-inflammatory activity of BRME was also measured on rat paw using arachidonic acid as the inducer. Arachidonic acid produces a short-lived oedema response characterized by a rapid onset and has been widely used to detect and evaluate lipoxygenase inhibitors and dual inhibitors of arachidonic acid metabolism in vivo [35]. Test extract showed marked inhibition of arachidonic acid-induced paw oedema in rats at all dose level, but lesser than indomethacin. The percent inhibition of all extract was utmost at 4 h (Table 4).

#### 3.3.4. Formalin-Induced Mice Paw Oedema

This experiment is a simple model of subchronic inflammation and used to screen antiarthritic and anti-inflammatory agents. The BRME produced significant (*P* < 0.05, 0.01 and 0.001) reductions in oedema development. The maximum effect was produced at 400 mg/kg on the 6th day (48.34%) when compared to others. The effect of indomethacin from 1st (43.49%) to 6th day (77.65%) was found significant (*P* < 0.001) (Table 5).

#### 3.3.5. TPA-Induced Mouse Ear Oedema

12-O-Tetradecanoilforbol-13-acetate, known as TPA, induces an acute inflammatory response, exemplified by vasodilation and oedema formation. This response occurs in the first two hours, followed by increased thickness of the ear. This inflammatory response is mediated by the PKC (protein kinase C) pathway which stimulates phospholipase A2 [36] and cyclooxygenase, resulting in the release, metabolism, and interaction of arachidonic acid and prostaglandin E2 [37]. Arachidonic acid metabolites such as PGI_2_ and LTB_4_ increase vascular permeability leading to oedema during the inflammatory [38]. Phospholipase A2 efficiently inhibits both leukocyte infiltration and oedema in the TPA model of ear inflammation [39].

Many compounds inhibit the enzymes such as LOX and COX that would be able to inhibit TPA induced inflammation. When TPA was applied topically to mouse ears, it promotes mast cell infiltration with release of mediators that increase vascular permeability and promote neutrophil influx [40]. TPA-induced local inflammation is more effectively controlled with arachidonate cyclooxygenase inhibitors than arachidonate lipoxygenase inhibitors. During the 1–4 h in TPA model ear oedema was increased due to increased vascular permeability, oedema, and swelling within the dermis.

**Figure 4:**
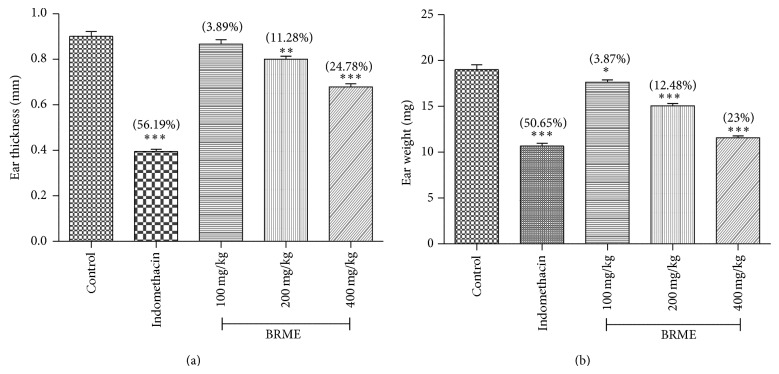
Effect of BRME and indomethacin on TPA-induced ear thickness (a) and ear weight (b). Values are expressed as means ± SEM (*n* = 6). ^*^ *P* < 0.05, ^**^ *P* < 0.01, and ^***^ *P* < 0.001 compared to the controls (one-way ANOVA followed by Dennett's test). (%) represents percentage inhibition of oedema development.

In the TPA-induced ear oedema, the BRME led to an inhibition of ear weight and thickness in a dose-dependent manner. The significant inhibition percentage was 11.28 and 24.78 in ear thickness and 12.48 and 23% for ear weight in 200 and 400 mg/kg of extract. Both of the extracts and indomethacin were found significant when compared to control (Figure 4).

#### 3.3.6. Acetic Acid-Induced Vascular Permeability

The main features of acute inflammation are vasodilatation, the exudation of plasma, increase of vascular permeability, and cell migration (primarily neutrophil) into the site of inflammation [41]. Increased vascular permeability occurs as a result of contraction and separation of endothelial cells at their boundaries to expose the basement membrane, which is freely permeable to plasma proteins and fluid [42].

Phlogistic agents increase vascular permeability at various times after injury in inflammation condition. Chemically-induced vascular permeability (such as seen with acetic acid) causes an immediate sustained reaction that is prolonged over 24 h and its inhibition suggests that the BRME may effectively suppress the exudative phase of acute inflammation [43].

**Figure 5:**
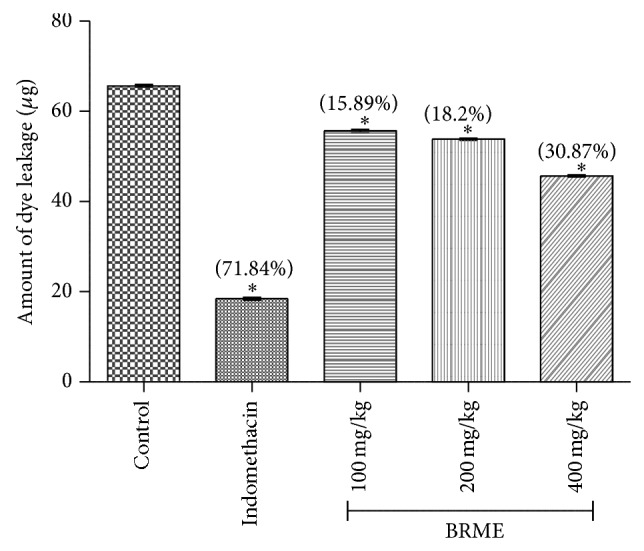
Effects of BRME and indomethacin in the acetic acid-induced vascular permeability. Values are expressed as means ± SEM (*n* = 6). ^*^ *P* < 0.05, compared to the control group (one-way ANOVA followed by Dennett's test). (%) represents percentage inhibition of dye leakage into peritoneum cavity.

The BRME reduces the dye leakage into the peritoneum in dose dependent manner. Reduction of dye leakage indicates its anti-inflammatory action due to reduced vascular permeability. In this method, the BRME extract on 100, 200, and 400 mg/kg and indomethacin (10 mg/kg) dose resulted in the significant (*P* < 0.05) inhibition of dye leakage by 15.89, 18.2, 30.87, and 71.84%, respectively (Figure 5).

#### 3.3.7. Total WBC Count in Paw Fluid

Carrageenan-induced increase in total WBC count occurs at the site of inflammation due to the release of IL-I inflammatory response, which increases the production of both granulocyte and macrophages colony stimulating factors [44]. The raised number of total WBC could mean there was either migration of WBCs to the site or increase in the population of the white cells preexisting at the site of inflammation. Leukocyte aggregation at the site of inflammation is a basic phenomenon in the inflammatory process [45].

Cell migration occurs as a result of much different process including adhesion and cell mobility. The increase in the total WBC count could be due to either migration of WBCs to the site or increase in the population of the white cells preexisting at the site of inflammation [9]. It is well established that indomethacin and other anti-inflammatory drugs inhibit migration of inflammatory cells by inhibiting the release of various chemical mediators [46].

**Figure 6:**
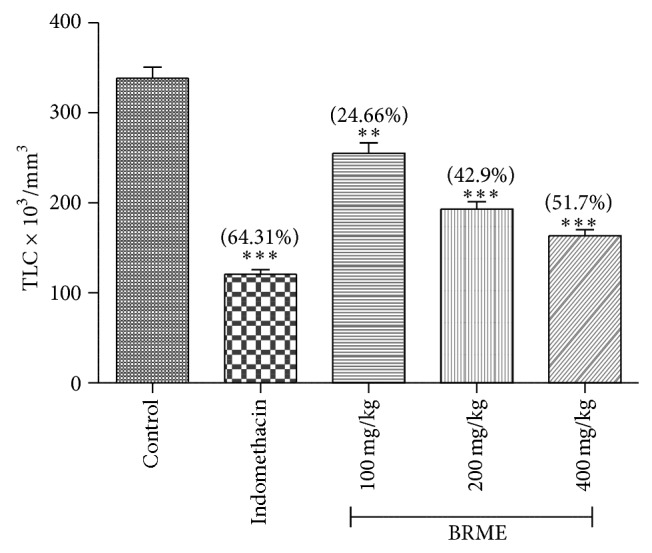
Effect of the BRME and indomethacin on the WBC count (×10^3^/mm^3^) in the paw fluid during acute inflammation induced by carrageenan. Each value represents the mean ± SEM (*n* = 6). Experimental groups were compared to control, ^**^ *P* < 0.01, ^***^ *P* < 0.001 (one-way ANOVA followed by Dennett's test). (%) represents percentage reduction of the total white blood cell count in paw fluid after inflammation.

In the present study, significant decrease in the WBC count caused by all doses of BRME and indomethacin suggests that these two agents inhibit the migration of WBCs to the site of inflammation. Reduction of the total white blood cell count in paw fluid after inflammation is induced by carrageenan by 24.66, 42.9, and 51.7% for the dose of 100, 200, and 400 mg/kg, respectively. Indomethacin also showed 64.31% inhibition (Figure 6).

#### 3.3.8. Myeloperoxidase Assay

Myeloperoxidase (MPO) is a highly oxidative enzyme standing as biochemical marker for tissue content of polymorphonuclear leukocytes (PMN) due to its correlation with the number of infiltrated cells in inflamed regions [47]. It is the most abundant proinflammatory enzyme released within the phagosome or the extracellular space from the neutrophil's azurophilic granules accounting for approximately 5% of their dry mass [48].

**Figure 7:**
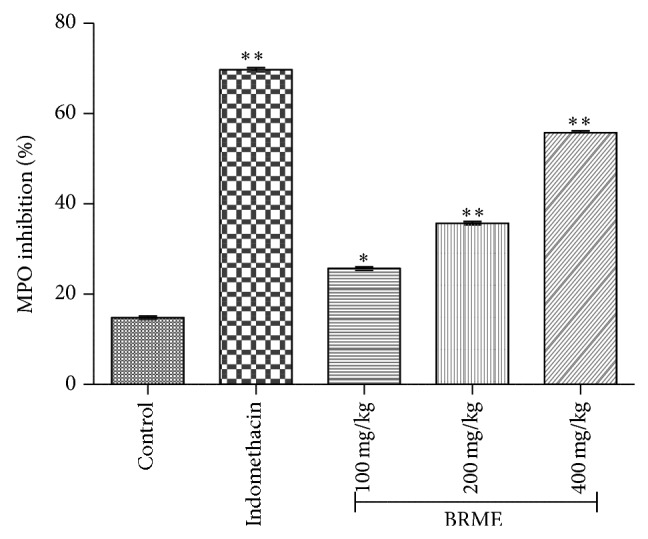
Effect of the BRME and indomethacin on % MPO inhibition. Each value represents the mean ± SEM (*n* = 6). Experimental groups were compared to control, ^*^ *P* < 0.05, ^**^ *P* < 0.01 (one-way ANOVA followed by Dennett's test).

MPO was also shown to promote lung neutrophilia and to influence indirectly subsequent chemokine and cytokine productions by other cell types in the lung. Increased intravascular levels of MPO are a hallmark of vascular diseases such as atherosclerosis sepsis, acute coronary disease, myocardial infarction, atrial fibrillation, multiple sclerosis, Alzheimer's disease, and ischemia and reperfusion, with levels reported to exceed 50 nM [49]. Thus, the reduction of MPO activity after the treatment of the animals with the evaluated extract, in both pleurisy and in the hind paw/ear oedema, is an indirect marker of a possible action for this extract on activated leukocytes. It could be suggested that MPO is also a marker of oxidative stress by generation of ROS. When it is released from defense cells, MPO acts on hydrogen peroxide formed by NADPH oxidase and increases the potential toxicity of this oxidant nM. MPO also promotes the generation of hypochlorous acid, a potent oxidant agent that is capable of inducing tissue lesions [50]. Here, we analyzed the effect of different concentration of extracts and indomethacin on TPA induced activity of MPO in the mouse ear oedema exudates (Figure 7), after a 4 h treatment.

In our study, we found marked reductions in the level of MPO enzyme activity on the homogenates from the inflamed tissues. Inhibitory activity for the dose of 200 (34.46 ± 4.2%) and 400 (55.13 ± 7.3%) mg/kg of extracts was found significant (*P* < 0.01) when compared to indomethacin (69.31 ± 5.6%), indicating high inhibition of neutrophil migration to the site of inflammatory (Figure 7). The results provide information about anti-inflammatory action of the extract being in part mediated by a reduction in the accumulation of neutrophils into the inflamed site.

## 4. Conclusion

Based on the results obtained from the study it can be concluded that* B. retusa* possesses significant anti-inflammatory potential and is also effective in inflammation associated pain. The present study indicates that methanol extract of* B. retusa* exhibited antinociceptive and anti-inflammatory properties, providing a scientific basis for its ethnobotanical uses for treating various ailments. This might be attributed, at least in part, to the presence of phenolic constituents. Analysis of BRME by HPLC and in vivo pharmacological methods identified phenolic compounds (gallic acid and ellagic acid) as major contributors to the anti-inflammatory activities of extract. Later on this knowledge could be applied to avert or suppress chronic inflammatory state. The many unknown phytoconstituents in the methanolic extract of* B. retusa* preclude inflammatory signaling. Hence an effort should be made to isolate and characterize the active agents responsible for the observed pharmacological activity.

## Figures and Tables

**Table 1:** Effect of methanolic extract of fruit of *Bridelia retusa* on carrageenan-induced paw oedema.

Treatment	Dose (mg/kg b.w.)	Increase in paw circumference (cm)				
		*T*_1 h_	*T*_2 h_	*T*_3 h_	*T*_4 h_	*T*_5 h_
Control		3.32 ± 0.05	3.78 ± 0.03	4.23 ± 0.06	4.42 ± 0.08	4.49 ± 0.06
Indomethacin	10	2.86 ± 0.03^***^ (49.27)	3.02 ± 0.06^***^ (55.65)	3.15 ± 0.05^***^ (60)	3.21 ± 0.06^***^ (60.89)	3.18 ± 0.04^***^ (63.98)
BRME	100	3.19 ± 0.05 (−1.45)	3.61 ± 0.06 (2.61)	4.03 ± 0.02 (3.75)	4.21 ± 0.03^*^ (3.91)	4.27 ± 2.63^*^ (4.3)
BRME	200	3.23 ± 0.03 (20.29)	3.56 ± 0.04^*^ (23.48)	3.89 ± 0.05^***^ (24.38)	4.02 ± 0.07^***^ (25.15)	3.93 ± 0.06^***^ (32.8)
BRME	400	3.08 ± 0.04^*^ (23.19)	3.37 ± 0.08^***^ (28.7)	3.59 ± 0.06^***^ (35)	3.66 ± 0.03^***^ (38.13)	3.62 ± 0.04^***^ (42.5)

Values are expressed as means ± SEM (*n* = 6). Figures in parenthesis indicate percentage inhibition of oedema development.

^*^ *P* < 0.05.

^***^ *P* < 0.001 (two-way ANOVA followed by Bonferroni's test).

**Table 2:** Effect of methanolic extract of fruit of *Bridelia retusa* on histamine-induced paw oedema.

Treatment	Dose (mg/kg b.w.)	Thickness of rat paw (cm)				
		*T*_1 h_	*T*_2 h_	*T*_3 h_	*T*_4 h_	*T*_5 h_
Control		2.64 ± 0.04	3.13 ± 0.05	3.64 ± 0.07	3.86 ± 0.06	3.98 ± 0.09
Indomethacin	10	2.31 ± 0.06^**^ (43.75)	2.47 ± 0.07^***^ (58.02)	2.53 ± 0.05^***^ (69.69)	2.57 ± 0.07^***^ (71.42)	2.59 ± 0.08^***^ (72.28)
BRME	100	2.59 ± 0.06 (3.13)	3.04 ± 0.06 (6.17)	3.39 ± 0.08^*^ (15.9)	3.52 ± 0.07^**^ (19.48)	3.58 ± 0.08^***^ (21.68)
BRME	200	2.57 ± 0.05 (9.38)	2.91 ± 0.04^*^ (22.22)	3.11 ± 0.07^**^ (37.12)	3.18 ± 0.09^***^ (41.56)	3.21 ± 0.09^***^ (43.98)
BRME	400	2.41 ± 0.05 (12.5)	2.73 ± 0.07^*^ (25.93)	2.91 ± 0.07^***^ (40.9)	2.97 ± 0.09^***^ (45.45)	3.01 ± 0.05^***^ (46.99)

Values are expressed as means ± SEM (*n* = 6). Figures in parenthesis indicate percentage inhibition of oedema development.

^*^ *P* < 0.05.

^**^ *P* < 0.01.

^***^ *P* < 0.001 (two-way ANOVA followed by Bonferroni's test).

**Table 3:** Effect of methanolic extract of fruit of *Bridelia retusa* on serotonin-induced paw oedema.

Treatment	Dose (mg/kg b.w.)	Thickness of rat paw (cm)				
		*T*_1 h_	*T*_2 h_	*T*_3 h_	*T*_4 h_	*T*_5 h_
Control		3.21 ± 0.03	3.64 ± 0.04	3.95 ± 0.02	4.11 ± 0.03	4.06 ± 0.06
Indomethacin	10	2.97 ± 0.04^***^ (45.71)	3.06 ± 0.06^***^ (64.1)	3.11 ± 0.03^***^ (69.72)	3.14 ± 0.05^***^ (71.2)	3.09 ± 0.04^***^ (74.17)
BRME	100	3.07 ± 0.06 (11.42)	3.42 ± 0.03^*^ (15.39)	3.57 ± 0.05^**^ (25.69)	3.66 ± 0.03^***^ (28)	3.59 ± 0.06^***^ (30.83)
BRME	200	3.08 ± 0.05 (14.29)	3.28 ± 0.02^**^ (35.9)	3.37 ± 0.03^***^ (45.87)	3.46 ± 0.04^***^ (45.6)	3.39 ± 0.05^***^ (49.17)
BRME	400	3.12 ± 0.04 (17.14)	3.29 ± 0.02^***^ (41.02)	3.41 ± 0.05^***^ (46.79)	3.46 ± 0.04^***^ (49.6)	3.39 ± 0.03^***^ (53.33)

Values are expressed as means ± SEM (*n* = 6). Figures in parenthesis indicate percentage inhibition of oedema development.

^*^ *P* < 0.05.

^**^ *P* < 0.01.

^***^ *P* < 0.001 (two-way ANOVA followed by Bonferroni's test).

**Table 4:** Effect of methanolic extract of fruit of *Bridelia retusa* on arachidonic acid-induced paw oedema.

Treatment	Dose (mg/kg b.w.)	Thickness of rat paw (cm)				
		*T*_1 h_	*T*_2 h_	*T*_3 h_	*T*_4 h_	*T*_5 h_
Control		2.76 ± 0.06	3.18 ± 0.07	3.57 ± 0.08	3.71 ± 0.06	3.59 ± 0.09
Indomethacin	10	2.02 ± 0.09^***^ (73.44)	2.11 ± 0.08^***^ (75.47)	2.18 ± 0.06^***^ (77.24)	2.22 ± 0.07^***^ (76.73)	2.16 ± 0.08^***^ (78.91)
BRME	100	2.51 ± 0.06 (1.56)	2.88 ± 0.04 (5.66)	3.02 ± 0.06^*^ (21.38)	3.11 ± 0.08^***^ (22.64)	3.07 ± 0.09^***^ (19.04)
BRME	200	2.46 ± 0.06^*^ (10.94)	2.67 ± 0.07^***^ (26.41)	2.84 ± 0.04^***^ (34.48)	2.91 ± 0.08^***^ (35.85)	2.87 ± 0.09^***^ (33.33)
BRME	400	2.43 ± 0.08^**^ (12.5)	2.64 ± 0.08^***^ (27.36)	2.73 ± 0.07^***^ (40.69)	2.77 ± 0.09^***^ (43.4)	2.71 ± 0.05^***^ (42.86)

Values are expressed as means ± SEM (*n* = 6). Figures in parenthesis indicate percentage inhibition of oedema development.

^*^ *P* < 0.05.

^**^ *P* < 0.01.

^***^ *P* < 0.001 (two-way ANOVA followed by Bonferroni's test).

**Table 5:** Effect of methanolic extract of fruit of *Bridelia retusa* on formalin-induced paw oedema.

Treatment	Dose (mg/kg b.w.)	Thickness of rat paw (cm)					
		Day 1	Day 2	Day 3	Day 4	Day 5	Day 6

Control		1.86 ± 0.15	2.12 ± 0.14	2.54 ± 0.12	2.78 ± 0.16	2.94 ± 0.17	3.13 ± 0.18
Indomethacin	10	1.37 ± 0.09^*^ (43.49)	1.43 ± 0.08^***^ (62.32)	1.54 ± 0.12^***^ (66.67)	1.56 ± 0.15^***^ (71.12)	1.58 ± 0.13^***^ (72.85)	1.55 ± 0.14^***^ (77.65)
BRME	100	1.67 ± 0.09 (4.65)	1.85 ± 0.12 (7.25)	2.21 ± 0.16 (9.9)	2.37 ± 0.13 (14.07)	2.46 ± 0.17^*^ (17.22)	2.68 ± 0.12^***^ (13.53)
BRME	200	1.54 ± 0.09 (6.98)	1.72 ± 0.06 (15.94)	1.98 ± 0.15^**^ (24.32)	2.13 ± 0.11^**^ (26.67)	2.24 ± 0.12^***^ (27.15)	2.39 ± 0.14^***^ (26.47)
BRME	400	1.52 ± 0.09 (20.93)	1.61 ± 0.1^*^ (37.68)	1.79 ± 0.13^***^ (45.04)	1.88 ± 0.11^***^ (48.04)	1.97 ± 0.12^***^ (47.68)	2.06 ± 0.12^***^ (48.34)

Values are expressed as means ± SEM (*n* = 6). Figures in parenthesis indicate percentage inhibition of oedema development.

^*^ *P* < 0.05.

^**^ *P* < 0.01.

^***^ *P* < 0.001 (two-way ANOVA followed by Bonferroni's test).
